# Advances in Smart-Response Hydrogels for Skin Wound Repair

**DOI:** 10.3390/polym16192818

**Published:** 2024-10-05

**Authors:** Yinuo Fan, Han Wang, Chunxiao Wang, Yuanhao Xing, Shuying Liu, Linhan Feng, Xinyu Zhang, Jingdi Chen

**Affiliations:** 1 Marine College, Shandong University, Weihai 264209, China; 15165524884@163.com (Y.F.); 15972359899@163.com (H.W.); wcx17661512175@163.com (C.W.); 13516335599@163.com (Y.X.); 19856132717@163.com (S.L.); 18953179141@163.com (L.F.); 17835855549@163.com (X.Z.); 2 State Key Laboratory of Mineral Processing, Beijing 100160, China; 3 Shandong Laboratory of Advanced Materials and Green Manufacturing, Yantai 265599, China

**Keywords:** hydrogel, smart response, chronic hard-to-heal wounds, trauma microenvironment

## Abstract

Hydrogels have emerged as promising candidates for biomedical applications, especially in the treatment of skin wounds, as a result of their unique structural properties, highly tunable physicochemical properties, and excellent biocompatibility. The integration of smart-response features into hydrogels allows for dynamic responses to different external or internal stimuli. Therefore, this paper reviews the design of different smart-responsive hydrogels for different microenvironments in the field of skin wound therapy. First, the unique microenvironments of three typical chronic difficult-to-heal wounds and the key mechanisms affecting wound healing therapeutic measures are outlined. Strategies for the construction of internal stimulus-responsive hydrogels (e.g., pH, ROS, enzymes, and glucose) and external stimulus-responsive hydrogels (e.g., temperature, light, electricity, and magnetic fields) are highlighted from the perspective of the wound microenvironment and the in vitro environment, and the constitutive relationships between material design, intelligent response, and wound healing are revealed. Finally, this paper discusses the severe challenges faced by smart-responsive hydrogels during skin wound repair and provides an outlook on the combination of smart-responsive hydrogels and artificial intelligence to give scientific direction for creating and using hydrogel dressings that respond to stimuli in the clinic.

## 1. Introduction

Factors such as accidents, assaults, surgeries, and diseases can cause severe damage to the skin, which in turn leads to the impairment of key functions [1,2]. There are typically four stages involved in the process of healing a skin wound. These stages are hemostasis, inflammation, repair, and maturation. During these stages, any disruption of the continuity of the skin or mucous membranes exposes deeper tissues to the risk of injury and infection, which ultimately results in the formation of chronic wounds [3]. Therefore, the rapid and effective healing of skin wounds, as well as the treatment of chronic hard-to-heal wounds, has always been a sought-after goal. The traditional means of wound healing mainly include natural composite therapy, surgical line suture, and traditional dressings [4,5]. There are many problems, such as long treatment cycles, scar retention, some treatments only having single functions, and the easy removal of secondary damage, that limit their application in the clinic [6].

In the context of a three-dimensional network, hydrogel is a polymeric substance that has the capacity to retain water. Its three-dimensional (3D) porous structure is similar to the natural extracellular matrix (ECM), which is why it has attracted a lot of interest regarding wound healing. Hydrogels that are synthesized from different raw materials have many different and individually beneficial properties, including hydrophilicity and water retention, self-healing, ductility, injectability, biocompatibility, biodegradability, and electrical conductivity [7,8,9,10]. These properties make hydrogels ideal for use in wound healing dressings as they allow for the on-demand shaping of the dressing at the wound site, the inhibition of bacterial growth, the absorption of wound exudate, resistance to inflammation, and the detection of wound characteristics [11,12,13].

The healing of wounds is an ongoing process that might involve many phases or types of chronic wounds involving different physiological response processes [14]. Therefore, the future direction of hydrogel dressings involves creating intelligent hydrogels that report abnormalities in the healing process by detecting the wound status and reactively adjusting to alterations within the wound microenvironment. Smart hydrogels, also known as stimuli-responsive hydrogels, alter the physical properties or chemical structure of the hydrogel molecule by responding to small changes in different internal (e.g., pH, enzyme, glucose, and ROS responses) and external stimuli (e.g., temperature, light, electrical, and magnetic field responses) and by reflecting the corresponding functions [15,16,17]. Examples include achieving precise drug release on demand and avoiding the drug resistance seen due to the use of high doses of antibiotics [18]. Therefore, when constructing hydrogel wound dressings, more and more researchers are focusing on the preparation of smart hydrogels and the realization of functionally responsive modulation through the introduction of substances capable of stimulating and responding to alterations in the wound’s microenvironment in order to meet the requirements of various stages of wound healing and various wounds that are healing.

**Scheme 1:**
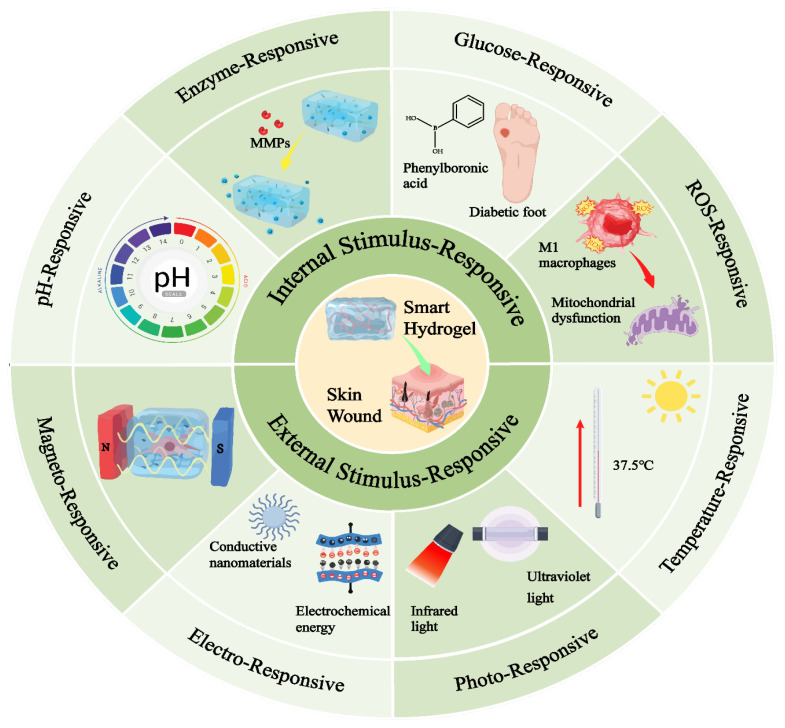
Classification and response principles of smart-responsive hydrogels applied in the field of skin wound healing.

This paper summarizes state-of-the-art cutaneous wound healing studies concerning smart-responsive hydrogel, mainly for chronic hard-to-heal wounds (Scheme 1). This article begins with a discussion of chronic wounds that are difficult to heal in cutaneous wounds. The topics covered include their microenvironment, physiological changes, and therapeutic measures. The primary focus of this discussion is on the intrinsic causes of the lengthy healing cycle of chronic wounds. Following this, the design principles and research findings of responsive hydrogels are discussed. These hydrogels are able to achieve functionally responsive modulation through the utilization of either internal stimuli (e.g., pH, blood glucose content, and enzyme concentration) or external stimuli (e.g., temperature, light, electricity, and magnetic field). Controlled drug release, achieved on demand through reversible or irreversible changes in chemical structures or physical properties to solve the problem of chronic wounds that are difficult to heal, has multiple stages, is difficult to regulate, and is extremely complex. In conclusion, the challenges for and developmental directions of smart-responsive hydrogel wound dressing, as well as future trends, are presented. The purpose of this presentation is to encourage the development of smart biomaterials in the future for the purpose of improving the management of chronic wounds that are difficult to heal and have complex pathologic microenvironments.

## 2. Microenvironment of Different Chronic Difficult-to-Heal Wounds and Therapeutic Measures

It is possible to describe chronic wounds that are difficult to cure as wounds in which the normal healing process is interrupted by a range of variables, both internal and external to the recipient, resulting in a pathologic inflammatory response that leads to delayed healing or the non-healing of the wounds. A number of factors can contribute to the development of chronic wounds [19]. Any factor that has an impact on the repair of wounds can cause the healing process to deviate from its typical sequence of steps, which in turn extends the amount of time it takes for the wound to heal [20]. The reasons for this can be divided into two broad categories: One category involves wound infection. Here, the immune cell activation is abnormal, resulting in a large number of inflammatory factors. Protein-hydrolyzing enzymes and reactive oxygen species are released. The tissue near the wound is in a state of excessive inflammatory response, meaning that the epidermis and granulation tissue cannot form for a long time. The other type involves wound ischemia and hypoxia, which reduce collagen synthesis and result in a large number of cell growth factors being degraded by abnormally activated matrix metalloproteinases, which limits the proliferation and migration of fibroblasts, epidermal cells, and so on, leading to the non-healing of the wound [21]. Currently, several common chronic difficult-to-heal wounds mainly involve drug-resistant bacteria-infected wounds, diabetic foot ulcers, burn wounds, and so on [22,23], and the microenvironments of and therapeutic strategies for these wounds are analyzed below.

### 2.1. Bacterially Infected Wounds

The role of bacteria in the maintenance of human health is significant and they are ubiquitous in nature. They control important aspects of immune system stability, cell proliferation and differentiation, and molecular metabolism. However, when these microorganisms are out of balance, they become pathogens and infect the organism [24]. The skin is susceptible to post-traumatic bacterial infections. The level of influence on wound healing is related to the type of bacteria, with common infecting microorganisms including *Staphylococcus aureus*, *Escherichia coli*, and *Pseudomonas aeruginosa*. The inflammatory period is the golden period for bacterial infection and proliferation. Therefore, infections caused by bacteria frequently accompany chronic wounds due to a long inflammatory period [25]. The prolongation of the inflammatory period will increase the release of pro-inflammatory cytokines, which will stimulate the release of proteases and inhibit the synthesis of protease inhibitors, and the overexpression of proteases will deplete growth factors and the extracellular matrix and also inhibit cell growth. Protease overexpression reduces cell proliferation by removing extracellular matrix and growth factors, leading to a vicious cycle of bacterial infection and prolonged inflammation. The long-term adhesion of bacteria results in the formation of biofilms (three-dimensional networks of bacteria in extracellular polymeric substances) with specialized channels for transporting nutrients, and the bacteria also undergo changes in metabolism and gene expression that increase resistance to drugs. Antibiotics are the mainstay of treatment for bacterial infections, but in recent years, the misuse of antibiotics has led to the problem of drug resistance and the emergence of ‘superbugs’, which are difficult to eliminate [26,27]. Currently, researchers are developing novel treatment options using antimicrobial materials or delivering antimicrobial drugs with biomaterials to increase the biosafety of the therapeutic process and reduce bacterial resistance. These materials mainly include film dressings, nanoparticles, hydrogel dressings, antimicrobial peptides, scaffolds, and so on [28,29,30,31,32,33].

### 2.2. Diabetic Foot Ulcer (DFU)

Patients with diabetes experience persistently elevated blood sugar levels, interfering with the regular biological activity of cells and thus affecting the normal healing of wounds and causing the formation of chronic ulcers. Diabetic foot ulcers are the most common chronic hard-to-heal wounds [34]. One of the most important factors in wound healing is reactive oxygen species (ROS). Low levels of ROS promote resistance to external microbial aggression, whereas a highly glycemic state produces large amounts of ROS, which imbalances the redox in the wound, increasing the difficulty of wound healing and prolonging the wound healing cycle [35,36]. Studies have shown that wound trauma in diabetic patients involves a highly oxidized microenvironment, which leads to a high expression of inflammatory factors, oxidative stress, the disruption of the extracellular matrix, dysfunction of the vascular endothelium, the inhibition of glyceraldehyde triphosphate dehydrogenase (GAPDH) activity, and the activation of signaling pathways related to diabetic complications. Not only that, but the high sugar microenvironment upsets the delicate equilibrium between TIMPs and MMPs [37,38]. Due to oxidative stress injury and the excessive release of inflammatory factors in the body, the concentration of MMP (MMP–2, MMP–8, MMP–9, MMP–14) increases and the concentration of TIMP (TIMP–1, TIMP–2) decreases, resulting in much larger MMPs/TIMPs than normal, leading to structural disturbances in the extracellular matrix. This affects fibroblasts, macrophages, keratin-forming cells, endothelial cells, and other cytokines’ normal biological functions, ultimately prolonging the inflammatory phase [39]. And unhealed ulcer wounds act as bacterial enrichment sites, where multiple bacterial infections exist. Bacterial growth disrupts the normal healing process, leaving ulcers unhealed and susceptible to scarring; even worse, prolonged hyperglycemia destroys the body’s immune system, leaving the body without the ability to clear the bacteria [40]. At present, the treatment of diabetic foot ulcer wounds is mainly divided into medical treatment and surgical treatment. Internal treatment is mainly performed through the use of relevant drugs to regulate the body’s blood glucose to normal values, and there is no substitute in the treatment of DFU. Infection control and surgical treatment are important measures that determine wound healing, limb preservation, and rehabilitation in patients with diabetic foot. Commonly used clinical surgical treatments for DFU include debridement, the negative-pressure closed-suction technique, antibiotic bone cement coverage, amputation surgery, topical adjuvant therapy, and stem cell therapy [41,42].

### 2.3. Burnt Wounds

Burns are an acute injury process, mainly caused by heat, cold, electric currents, radiation, and chemicals, and can be classified as first-, second-, third- and fourth-degree burns according to the depth of the skin involved and the degree of damage [43]. The skin is composed of three parts: the epidermis, the dermis, and the subcutaneous tissue [44]. The epidermis is very thin and consists of five layers: the stratum corneum, stratum pellucidum, stratum granulosum, stratum spinosum, and stratum basale [45]. The cells between the layers are small, organized, and tightly packed. The epidermis is waterproof, prevents tissue fluid from flowing out, is resistant to abrasion, and resists infection. The dermis is relatively loose and contains many skin appendages, such as collagen fibers, hair follicles, blood vessels, and lymphatic vessels. The thickness of the dermis and the amount of fibrous tissue and matrix are closely related to the firmness, fullness, laxity, and wrinkles of the skin. The subcutaneous tissue is located between the dermis and the connective tissue, being composed of loose connective tissue and fat lobules. It is connected to the dermis in the upper part and to the fascia, tendon membrane, and periosteum in the lower part. The subcutaneous tissue has the function of connectivity, the cushioning of mechanical pressure, energy storage, and warmth preservation [46]. First-degree burns only involve the skin’s outermost stratum. The local skin surface is erythematous, and there is a burning sensation. These burns occur without blisters, and can heal on their own without leaving scars [47]. Deep second-degree burns and superficial second-degree burns are the two main categories of second-degree accidents [48]. Superficial second-degree burns injure the epidermis and part of the dermis, and heal with a small amount of pigmentation and no scarring [49]. Deep second-degree burns to the dermis cause susceptibility to infection, blistering, significant pigmentation after healing, and scarring. Third-degree burns fully penetrate all layers of the skin; fourth-degree burns can extend to all layers of the skin and even deep into muscles and organs [50]. Burns above the third degree require surgical skin grafting, while burns of the first and second degree can be treated with herbal products, new medications, and biological dressings to enhance the healing of burn wounds. There are several important stages in the healing process of burn wounds, such as coagulation, inflammation, vascular regeneration, epithelialization, contraction, and remodeling [51]. The biggest difference between burns and other wounds is that blood vessels are damaged, restricting or terminating blood flow to the wound site. Coagulation, which involves the narrowing of blood vessels and the secretion of growth factors by various cells such as macrophages and fibroblasts, is the initial stage of the healing process [52]. In addition, during the skin regeneration process, cells such as fibroblasts, keratinocytes, and mesenchymal stem cells are active and produce various cytokines, such as IL–7, IL–8, IL–10, IL–12, TNF–α, vascular endothelial growth factors (VEGFs), and CD surface markers, to promote angiogenesis and stabilization [1].

## 3. Internal Stimulus-Responsive Hydrogels

The design of intrinsic internal stimuli for different chronic difficult-to-heal wounds is crucial due to the fact that different pathological microenvironments exist in different wounds (Table 1). These internal stimuli can specifically correspond to different pathological microenvironments, which in turn improves the targeting and accuracy of wound healing [53]. As wound dressings continue to evolve, new materials and smart responses are beginning to emerge, allowing us to build specific response products for precise healing. To our knowledge, internal stimulus-responsive hydrogels that can be used for skin wound healing are mainly categorized into chemostimulatory (involving pH and ROS) and biostimulatory (involving enzymes and glucose) variants [54,55,56,57].

### 3.1. pH-Responsive Hydrogels

**Figure 1:**
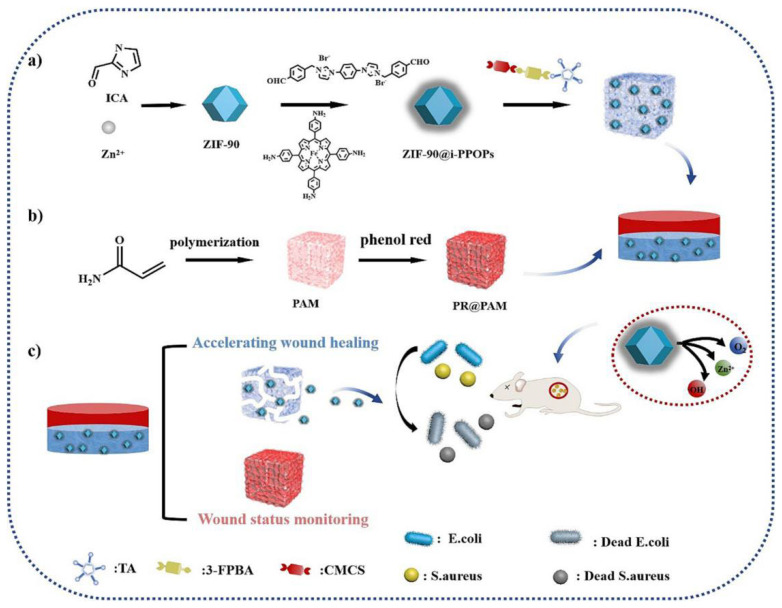
A schematic illustration of the preparation process of ZIF-90@i-PPOP–phenol red-based double-layered hydrogels for wound status monitoring and the acceleration of wound healing [75].

The normal pH range for skin is 4–6, but inflammatory exudates from wounds can cause the pH to be higher than normal, which affects cellular metabolism within the wound bed. But when wounds are still in the debridement and healing phases, the pH often changes to a more acidic state; as the healing process nears its end, an alkaline environment is required due to the proliferation of keratinocytes and fibroblasts [74]. The pH in the wound might change based on the stage of healing and any accompanying infection. Bacterial metabolism is known to generate acidic byproducts such as malic acid, lactic acid, and acetic acid. As a result, the infection site has an acidic microenvironment. Li et al. [75] designed multifunctional bilayer hydrogels with pH stimulus-responsive behavior for the purpose of smart real-time infection monitoring and wound care, utilizing tannic acid and carboxymethyl chitosan. In this system (Figure 1), the zinc-based zeolite imidazolium backbone @ peroxidase (ZIF–90@i–PPOPs) was engineered into an inner hydrogel through a Schiff base reaction between biomolecules, tannins, carboxymethyl chitosan, and 3-formylphenylboronic acid, and the outer hydrogel consisted of polyacrylamide (PAM, a biocompatible cationic polymer) and phenol red, forming a pH sensitivity-based color developer composition. It is the stimulation that causes the inner hydrogel to break down, which ultimately leads to the precise delivery of positively charged ZIF–90@i–PPOPs to the site of the injury. This is because the microenvironment within the wound is weakly acidic. Due to the gradual degradation of ZIF-90 in acidic environments, the released zinc ions can participate in antimicrobial therapy through direct cell membrane deformation, and the i-PPOPs have the required catalytic efficiency in acidic environments to catalyze the generation of O_2_ from H_2_O_2_, which can be utilized to scavenge reactive oxygen species. At the same time, the outer hydrogel can be monitored in real time for changes in pH to assess wound recovery. Plus, in vivo trauma models demonstrated that the hydrogel has good biocompatibility, good tissue adhesion, good bacterial eradication capabilities, and good wound healing properties.

The appropriate regulation of the pH microenvironment of diabetic wounds, according to reports, speeds up the healing process of wounds. Xia et al. [76] constructed a multifunctional hydrogel that continuously operates. In order to provide a fresh approach to the treatment of diabetic wounds, this method intelligently adjusts the pH of the wound microenvironment. The researchers designed a glycopeptide-based hydrogel (HPADN hydrogel), consisting of ammoniated or aldehyde-functionalized hyaluronic acid, and modified poly (6-aminohexanoic acid) containing strontium ionic dopamine. This dressing is able to modulate the pH microenvironment by protonating and deprotonating adjacent nonpolar structures in a neutral microenvironment. The use of HPADN hydrogels changes the pH of the wound site from the previous pH of approximately 7.0 to one of approximately 6.5. The polarization of macrophages to the M2 type, which contributes to the reduction in inflammation, occurs inside this surrounding milieu. In addition to this, endothelial cells have the ability to undergo fast vascularization, which contributes to the reduction in hypoxia in the surrounding environment. During the period of proliferation, the pH of the environment that surrounds the wound becomes roughly 7.0, which is an increase from the previous value of 6.5. At this point in time, the newly formed blood vessels supply an adequate amount of nutrients for the proliferation of fibroblasts, and the microenvironment is neutral, which does not have any impact on the proliferation of fibroblasts. The results from in vivo experiments confirmed that the hydrogel speeds up the healing process for diabetic wounds and encourages the growth of new blood vessels, which aids in skin regeneration.

### 3.2. ROS-Responsive Hydrogels

The phrase reactive oxygen species (ROS) is an umbrella term for oxygen-containing and radical-forming peroxides associated with oxygen metabolism in living organisms, including singlet oxygen (^1^O_2_), hydrogen peroxide (H_2_O_2_), superoxide anion (O_2•_^−^), and the hydroxyl radical (·OH) [77,78]. ROS are involved in recruiting immune cells and destroying bacteria and perform a crucial function in promoting wound healing. On the contrary, an excessive amount of ROS can result in oxidative stress [79], causing damage to proteins and DNA, which can slow down the healing process of wounds. ROS-responsive hydrogels are able to detect oxidative stress in the surrounding environment. A responsive bond-breaking process results in the release of the drug from the hydrogel and prevents the production of an excessive amount of reactive oxygen species. Phenylboronic acid ester (PBAE) is a structure that is extremely vulnerable to ROS due to the cleavage of the C–B bond in PBAE at the pathological level of ROS [80]. For example, Wu et al. [81] developed an injectable glycopeptide hydrogel using phenylboronic acid (PBA) and dextran oxide (POD) as its substrates. In this case, the boronic acid group of the POD chain crosslinks with the catechol group in the caffeic acid-modified poly lysine chain to form a boronate bond, which breaks during the healing process of infected diabetic wounds to induce the anti-inflammatory activity of diclofenac sodium (DS), thereby exerting its anti-inflammatory effects. Zhu et al. [82] utilized the rapid formation of borate esters, based on TSPBA (PBA derivative, ROS-responsive crosslinker), between TSPBA (PBA derivative, ROS-responsive crosslinker) and polyvinyl alcohol (PVA) for the co-delivery of metformin and fibroblast growth factor 21 (FGF21) to efficiently scavenge ROS and promote wound healing.

### 3.3. Enzyme-Responsive Hydrogels

Derived from the observed behavior of site-specific enzymes, enzyme-responsive hydrogels enable chemical bond breaking or the polymer degradation of hydrogel materials. In the pathologic process, several enzymes are crucial. Of these, proteases are the most widely studied enzymes [83]. Proteases and their inhibitors control the degradation and deposition of ECM, two processes that are essential to nearly every step of wound healing. In particular, the enhanced expression of ECM remodeling enzymes (e.g., MMP), highlighted in the case diabetic wounds, leads to the degradation of various protein-based growth factors, which ultimately delays wound healing [84]. As a solution, scholars have looked into MMP-responsive hydrogel-based drug delivery systems to improve wound healing rates in chronic wounds. For instance, Li et al. [85] developed MMP-responsive hydrogels loaded with deferoxamine (DFO). In these, MMP cleaves peptide-grafted hyaluronic acid and then crosslinks oxidized dextran to form a gel via Schiff base reaction, which intelligently releases DFO in diabetic wounds in rats, thereby enhancing the production of hypoxia-inducible factor–1α (HIF–1α). This speeds up the processes of blood vessel formation and healing after injury. Furthermore, based on gelatin being an intrinsic degradation substrate of MMP–9, Zhou et al. [86] promoted adequate cellular uptake of INS and Cel by encapsulating insulin (INS) and gelatin microspheres containing celecoxib (GMs @ Cel) in a hydrogel network, which released Cel on demand in the environment of MMP–9, in addition to modulating the local expression level of MMP–9 in wounds to accelerate chronic diabetic wound healing. Take note of the fact that oxidoreductase-induced catalyzed free radical polymerization into hydrogels primarily uses horseradish peroxidase (widely derived from plants) and hydrogen peroxide-catalyzed systems [87], which are primarily introduced exogenously and therefore are not part of the internal environmental stimulus-responsive hydrogels.

### 3.4. Glucose Responsive Hydrogels

**Figure 2:**
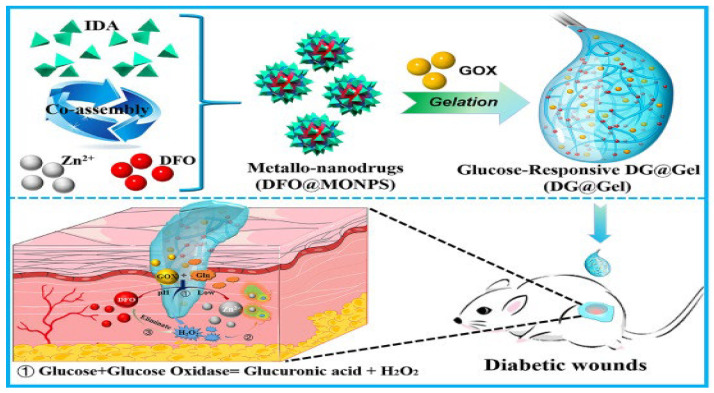
Schematics of the preparation of glucose-responsive metal–organic hydrogel and its working mechanism in terms of repairing diabetic skin wounds [91].

It subsequently appears that the development of glucose-responsive hydrogels could be of assistance in the management of diabetic wounds. This is because diabetic patients present with high blood glucose levels. The three most common methods for creating hydrogels that respond to glucose levels are the reversible binding of phenylboronic acids (PBAs) to glucose hydroxyl groups, the action of glucose oxidase (GOX) to convert glucose into gluconic acid, and the specific recognition of glucose by proteins that bind to glucose [88,89]. GOX is an enzyme that converts glucose into gluconic acid. PBAs and glucose can be reversibly bound together, which is the most common method among these. If one wants to create hydrogels that react to changes in glucose levels, this is the method that offers the greatest potential. For example, Xu et al. [90] prepared glucose-responsive hydrogels to scavenge high levels of ROS from diabetic wounds using PBA-modified hyaluronic acid (HA–PBA). By creating a dynamic borate bond between the polyphenol moiety of populin (MY) and the phenylboronic acid moiety of HA-PBA, the hybrid hydrogel was able to immobilize the MY molecule, which had significant antioxidant activity, and the high content of glucose at the wound site caused the binding of the phenylboronic acid moiety, which triggered the release of MY intelligently. Furthermore, Yang et al. [91] developed a hydrogel that contains a medication and responds to glucose levels by GOX loading (Figure 2). GOX present in the drug-carrying gel breaks down excess glucose into hydrogen peroxide and glucuronic acid, thereby lowering the pH at the wound site, with the low pH promoting the release of zinc ions and DFO, which have synergistic antimicrobial and angiogenic activities for diabetic wound repair. Glucose-binding proteins are typified by companion Concanavalin A (Con A), and upon the binding of free glucose molecules to hydrogels functionalized with Con A, a reversible sol-gel phase change occurs in the polymer network, enhancing insulin release from the gel system [92].

## 4. External Stimulus-Responsive Hydrogels

In contrast to the chemical and biological stimuli responses of the internal stimulus-responsive type, the external stimulus-responsive type mainly achieves on-demand release and spatiotemporal modulation by utilizing physical stimuli [93]. The long-term stability of external stimulus-responsive hydrogels is related to a variety of factors. For example, factors like temperature, pH, and ROS may affect the stability of hydrogels [94]. Hydrogels may freeze at low temperatures, lose water in dry environments, and absorb water and swell in conditions of high humidity. They may disintegrate at low pH values and high ROS activity to release drugs, nanoparticles, microspheres, actives, etc., for the purpose of accelerating wound healing. Secondly, the crosslinking density of hydrogels has a significant effect on their stability. By increasing the crosslinking density, the mechanical strength and stability of hydrogels will be relatively improved. Not only that, different polymers also have different stabilities [95]. For example, polyvinyl alcohol (PVA) is a typical physical gel material, which can be frozen and thawed to obtain a stable hydrogel at a certain temperature. Its degradation rate is relatively slow. The use of specific solvent systems, such as glycerol–water hybrid systems, can also enhance the freezing resistance and heat resistance of hydrogels, thus improving their long-term stability [96]. In summary, the long-term stability of external stimulus-responsive hydrogels requires the comprehensive consideration of various factors, which we can realize through fine design and preparation methods. External stimulus response strategies for traumatic wound healing mainly involve temperature, light, electricity, and magnetic fields. Different physical stimulation strategies involve different response mechanisms to external conditions (Table 2).

### 4.1. Temperature-Responsive Hydrogels

#### 4.1.1. Thermoresponsive Hydrogels

Thermoresponsive hydrogels are of interest in wound dressings because they gel at physiological temperatures, allowing them to assume the shape of asymmetric wounds. Thermoreversible temperature-responsive gels fall into two broad categories. The first category relies on a hydrophilic/hydrophobic balance, such as Pluronic F127, where temperature increases the hydrophobic effect, driving the micelle formation, modifying the shape of the micelles, and enhancing the contact between the micelles. This ultimately results in the formation of gels of the substance. The second category of thermoreversible gel calls for the presence of a temperature-responsive polymer component that, when subjected to a change in temperature, initiates the process of self-assembly. Poly (N-isopropylacrylamide) (PNIPAM) is a typical thermoresponsive polymer that is widely used in human-related medical treatments due to its lower critical solution temperature, which is close to the human physiological temperature. For example, Cai et al. [110] developed a temperature-sensitive multifunctional smart hydrogel patch containing PNIPAM. Upon the wound application of the hydrogel, because of its temperature sensitivity, the hydrogel’s internal phase shifted, making it more contractile and hence better able to aid in wound healing. Zhou et al. [111] improved the capability of mice to heal full-thickness skin wounds by topically applying a complex that consisted of injectable, biocompatible, and thermosensitive Pluronic F127 hydrogel-encapsulating exosomes generated from human adipose mesenchymal stem cells. Reduced inflammation, increased collagen deposition, and enhanced wound re-epithelialization are all possible outcomes of using hydrogel.

Evidence suggests that accelerated enzymatic reactions at the site of an infected lesion cause a temperature increase (often between 1 and 4 degrees Celsius) in the affected area and the surrounding area. Therefore, by employing temperature fluctuations, it is possible to develop hydrogel wound dressings that are temperature-responsive and have regulated medication release. In one study, temperature-sensitive hydrogels containing a minocycline hydrochloride (MH) complex were designed to treat infected wounds. Poroxam 407 (P407) and Poroxam 188 (P188) were used as the main gel carriers to encapsulate the MH and Ca^2+^ complexes. As a consequence of this, the wound was protected by a solid gel that was highly shape-adaptive to the wound, and this solid was filled with fluid gel in order to provide the necessary filling. It is also important to note that the complexation of MH with Ca^2+^ results in the gradual release of MH, which serves the purpose of promoting wound repair in infected areas and also provides antibacterial and anti-inflammatory treatment [112]. In an ideal scenario, the therapeutic drug release from the temperature-sensitive gel begins when the gel reaches the target temperature, and it stops when the gel reaches the target healing temperature and the temperature returns to normal. This occurs when the warmth of the wound rises above the normal core temperature.

#### 4.1.2. Cold Responsive Hydrogels

**Figure 3:**
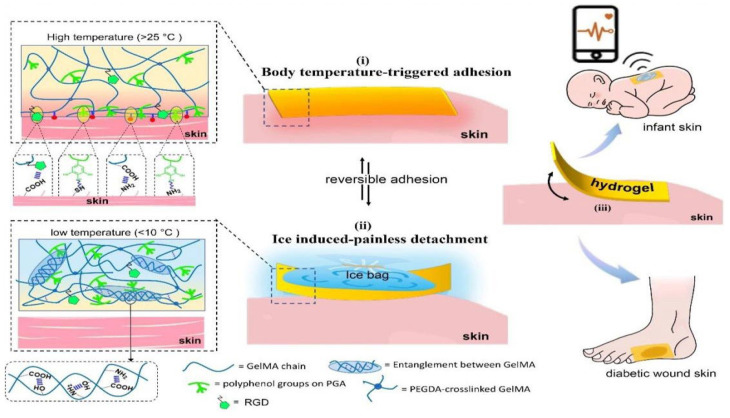
Design strategy for skin-friendly hydrogel patches with body temperature-triggered fast adhesion and non-destructive on-demand peeling [113].

Motivated by hydrogen bonding’s reversibility at different temperatures, Jiang et al. [113] utilized the multiple interactions of polyphenol groups to design a temperature-sensitive skin-friendly hydrogel patch (Figure 3). When the hydrogel was in contact with human skin, the body temperature triggered the phase transition to achieve efficient and rapid adhesion and to keep the adhesion stable for a long time. After a simple cooling of the hydrogel surface, the molecular chains in the network regenerate hydrogen bonds, and the hydrogel adhesion decreases, realizing non-destructive peeling. In addition, the hydrogel patch gently adheres to the injured skin, providing a favorable environment for accelerating the healing process and thus controlling diabetic wounds.

### 4.2. Photoresponsive Hydrogels

#### 4.2.1. UV-Responsive Hydrogels

Ultraviolet (UV) light has a short wavelength, offering the lowest tissue penetration but the highest energy. UV-modulated smart hydrogels are commonly used for rapid sol–gel transformations and as bioadhesive materials. The traditional method involves the formation of free radical-polymerized hydrogels of double-bonded polymers under photoinitiated polymerization. For example, Ding et al. [3] prepared a multifunctional wound hydrogel dressing based on gelatinized methacrylate (Gel MA) by UV irradiation with ease. Wang et al. [114] used vinyl to chemically modify hyaluronic acid (HA), dextran (Dex), and β–cyclodextrin (β–CD) to generate biocompatible hydrogels with enhanced mechanical strength under UV irradiation. They found that vascular endothelial growth factor (VEGF) plasmids accelerated the healing of splint-excised burn wounds, in particular via enhancing microvessel development and suppressing the inflammatory response, while also being biocompatible. In addition, Zou et al. [115].grafted methacrylate and catechol groups onto the backbone of carboxymethyl chitosan (CMCS), grafted aldehydes and borophenyls onto the backbone of sodium alginate (SA), and tannic acid (TA) was added to the hydrogels. Three types of covalent bonds-dynamic, light-triggered, and hydrogen-bonding-underpin this. Hydrogels with higher strength, flexibility, and bio adhesion were formed by multiple crosslinking. The efficiency of the hydrogel in healing infected wounds was significantly better than that of the commercially available gel dressings.

However, the free radical-mediated photo crosslinking curing mechanism has never been able to circumvent the free radicals, bringing biotoxicity, and oxygen barrier defects [116]. Zhu’s team was the first to propose a ‘photocoupling reaction’ as a non-radical crosslinking mechanism. In this, different types of onitrobenzyl compounds were grafted onto biomolecules, and under UV light irradiation, the onitrobenzyl ester mechanism could be photolyzed into carboxylic acid and onitrobenzylidene acetaldehyde. The photo-induced aldehyde group is covalently bonded with the amino group on the tissue surface, and photo in situ gel technology with tissue adhesion and integration characteristics has been established, which solves the problem whereby the traditional ‘soft and smooth’ hydrogel cannot adhere and be fixed to humid and dynamic tissue surfaces. In particular, the team used cyclic onitrobenzyl compound-modified hyaluronic acid to develop a bio-glue that cures within 5 s. When exposed to UV light, cyclic onitrobenzyl compounds release sulfhydryl, nitroso, and aldehyde groups, where sulfhydryl crosslinks with nitroso, and aldehyde combines with the amino group of the protein on the surface of the tissue to realize the adhesion and immobilization of the adhesive to the tissue [117].

Azobenzene has photoisomerization properties (trans and cis isomers), and UV light induces trans to cis isomerization, thereby modulating the crosslinking density of supramolecular hydrogels. Based on this concept, Zhao et al. [118] prepared light-responsive supramolecular polysaccharide hydrogels via the host–guest interaction between azobenzene and hyaluronic acid chain-coupled β-cyclodextrin moieties. Under UV conditions, the hydrogels transformed into a relaxed network structure, thereby rapidly releasing epidermal growth factor (EGF). This enhanced EGF delivery at the wound site and demonstrated increased levels of growth factors, granulation tissue, and angiogenesis, all of which contribute to better wound healing efficiency.

#### 4.2.2. Visible Light-Responsive Hydrogels

Visible light-induced photopolymerization provides greater tissue penetration, deeper tissue penetration, and safer irradiation than UV light. Currently, photopolymerization is mainly achieved using UV. This may lead to cellular damage during exposure; however, when visible light supersedes ultraviolet light, photosensitized hydrogel systems can be realized with higher cytocompatibility and broader application prospects. Visible light cross linkable hydrogels are mainly dependent on visible light-initiated free radical polymerization. There are various types of free radical photo initiators, which are mainly classified into Type I (photo initiators involving one-component pyrolysis) and Type II (photosensitizing/co-initiator photo initiators). Type I typically uses lithium phenyl-2,4,6-trimethylbenzoyl hypophosphite (LAP, visible light polymerization at 400 nm). Type II mainly uses ruthenium pyridine complexes (visible polymerization at 400–700 nm), eosin Y (visible polymerization at 470 nm), fluorescein (the most commonly used excitation wavelengths are 488 nm and 633 nm), and camphorquinone (visible light in the 400–500 nm range) [119]. Among them, the visible light photosensitizer eosin Y has been shown to be a safe photo crosslinking system for biomedical applications because of how well it dissolves in water, its absorption peak at about 515 nm, and its low cytotoxicity [120]. Guo et al. [121].utilized a hemostatic bio adhesive (HAD) prepared from thrombin and Gel MA. When exposed to visible light, the use of eosin Y in free radical polymerization allows for the production of HAD. The photo crosslinking of HAD in bleeding tissues under visible light (430–530 nm) conditions can form a physical barrier and efficiently convert fibrinogen into fibrin for rapid hemostasis and the sealing of tissues.

**Figure 4:**
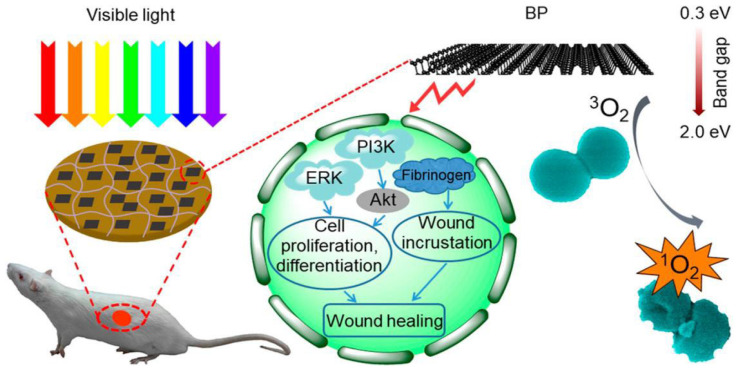
A schematic illustration of the BP hydrogel-based accelerated healing of infected wounds under visible light irradiation [122].

In addition, the irradiation of photosensitizers at visible wavelengths effectively kills bacteria through energy transfer and the generation of free radicals. Organic photosensitizers mostly consist of macrocyclic molecules, such phthalocyanines, indocyanine dyes, phenothiazines, and porphyrins, whereas zinc oxide, titanium dioxide, black phosphorus, graphene, and MXenes belong to the inorganic-based photosensitizers. For example, a composite hydrogel embedded with two-dimensional black phosphorus nanosheets (BPs) was prepared by Mao et al. [122]. Within 10 min under simulated visible light, the composite hydrogel exhibited a strong bactericidal effect against 98.90% of *Escherichia coli* and 99.51% of *Staphylococcus aureus* due to a strong ability to generate linear oxygen, accelerating the healing of infected wounds by accelerating the regenerative activity of skin cells and eradicating bacteria (Figure 4). Furthermore, nearly 99.99% of *Staphylococcus aureus* and multidrug-resistant *Staphylococcus aureus* were eradicated in vitro under mild light irradiation using a hybridized hydrogel that consisted of sodium porphyrin in porphyrin photosensitizer and poly (lactic acid–glycolic acid) (PLGA)-encapsulated basic fibroblast growth factor (BFGF) nanorods embedded in CMCS-SA. Using a model of burn infection, the multifunctional hybridized hydrogel effectively reduced bacterial growth and enhanced wound healing [123]. Methacrylic acid-based hydrogels are common click chemistry photoresponsive hydrogels. Researchers have used molecular dynamics simulations to analyze the thermal properties and mechanical stability of methacrylic acid porous hydrogels in different environments, saving labor and time costs and gaining the ability to compute optimal solutions with high performance [124].

#### 4.2.3. Infrared Light-Responsive Hydrogels

Infrared light has a much longer wavelength and is characterized by excellent tissue penetration depth, high stability, and low irritation. Currently, smart hydrogels based on the response of near-infrared light (NIR, 750–2500 nm) are often utilized to promote wound healing. They do so by exhibiting photothermal activity (e.g., killing bacteria at the wound site or promoting blood circulation) as well as by generating gases (CO improves microcirculation and increases the local oxygen level, and NO promotes angiogenesis and affects the immune response) [125,126].

**Figure 5:**
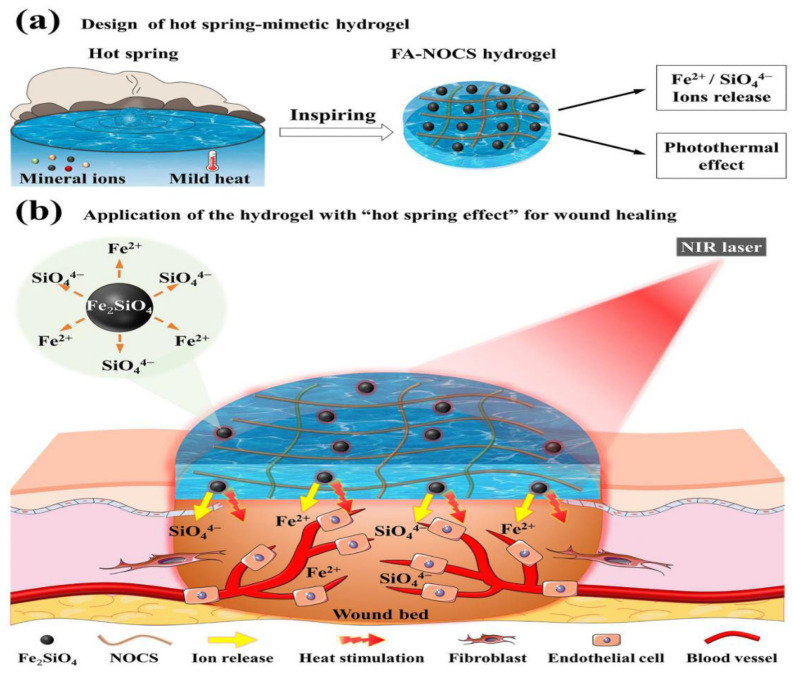
The scheme of the hot spring mimetic hydrogel for wound healing application [128].

Photothermal treatment (PTT) offers great promise in terms of targeting specific areas with heat. Recently, the most common photothermal agents utilized in the synthesis of NIR-responsive photothermal agents have been transition metal sulfide/oxide nanomaterials (e.g., CuS, TiO_2_, and MnO_2_), metal nanostructures (e.g., Cu NPs, Ag NPs, and Au NPs), carbon-based materials (e.g., carbon nanotubes and graphene and graphene oxide), conjugated polymers (e.g., polypyrrole, PDA, and polyaniline), iron-catechol complexes, and Prussian blue nanoparticles, among others. Zhao et al. [127] developed a photothermally active dual-network hydrogel for the treatment of wounds infected with multidrug-resistant bacteria. The hydrogel responds to light by coordinating with metal ions of catechol and iron, which raises the temperature of the hydrogel during near-infrared irradiation, thereby killing the bacteria at the wound site and therefore preventing the infection of the wound. Also, the idea came from the practical use of hot springs. Sheng et al. [128] designed a novel bioactive photothermal hydrogel. The hydrogel releases bioactive ions and has a heating function in order create a mildly heated and bioactive ion (Fe^2+^ and SiO_4_^4−^) environment in the wound area, showing significant enhancement in terms of promoting angiogenesis and chronic wound healing (Figure 5).

In addition, combining the photothermal effect and light-induced gas generation to play a multifunctional role, Liu et al. [129] encapsulated BNN6, a donor of NO which was loaded into zeolite imidazolium framework-8 (ZIF-8) modified with polydopamine (PDA), inside a hydrogel composed of gelatin and levoglucinic anhydride. The PDA enhanced the photothermal effect of the nanoparticles, and at the same time, the BNN6 was able to, under the irradiation of 808 nm NIR-stabilized NO release, greatly promote the healing of bacteria-infected wounds and significantly enhance angiogenesis. These factors also inform the design of multifunctional dressings that make full use of NIR.

### 4.3. Electro Responsive Hydrogels

One of the skin’s inherent properties is electrical conductivity, which speeds up the healing process by promoting keratinocyte migration, enhancing epithelial regeneration, directing angiogenesis, and modulating the expression of various factors. Conventional electrical stimulation (ES) methods do not cover the entire wound area and thus are detrimental to the wound healing process. Currently, the use of electroactive scaffolds combined with the application of electric fields to accelerate skin regeneration at the wound site is an attractive adjunct to wound care [130]. A conductive hydrogel wound dressing is one of the most common types of electroactive scaffold. It creates a physical barrier between the wound and the external environment, preventing microorganisms from invading the wound and reducing the risk of infection. Conductive hydrogels mostly contain an electroactive substance that covers the wound area and comes into contact with the skin underneath the wound, thus creating a circuit. This circuit efficiently transmits electrochemical and electro-biological signals to the cells within the affected tissue, resulting in a series of therapeutic responses. Lei et al. [131] prepared dynamically crosslinked conductive hydrogel dressings that fit perfectly into the cavity of deep wounds. In addition, the combination of exogenous ES therapy promotes cell-to-cell signaling and current transfer from external electrical stimuli, thereby promoting cell migration and angiogenesis.

**Figure 6:**
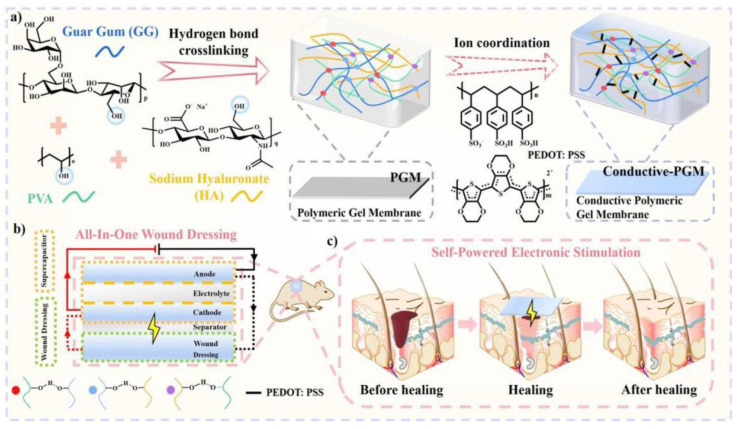
A schematic diagram of the electronically stimulated healing process of a self-powered integrated wound dressing [132].

Nevertheless, in order to supply the appropriate electric field, practical clinical applications typically require the utilization of large external power supply devices. This results in a significant amount of inconvenience for the healing process. Wang et al. [132] designed and prepared a wound dressing that integrates a supercapacitor and a wound dressing. During the course of the electrical stimulation treatment, the supercapacitor located in the upper layer is able to supply the wound dressing located in the lower layer with electrical energy so that the patient can enjoy the benefits of electrical stimulation treatment whenever and whenever they like, thus broadening the use scenarios of ES treatment (Figure 6). On the other hand, the unique one-piece wound dressing incorporates odium hyaluronate into both the upper and lower layers. Applying ES to the wound dressing below causes the release of sodium hyaluronate, an active ingredient that has the ability to enhance wound healing by speeding up the healing process and promoting the multiplication of fibroblasts.

Conductive hydrogels can be divided into ionic conductive and conductive polymers. These are commonly dispersed inside the gel with metal nanoparticles, polyaniline, polypyrrole and polythiophene, graphene and carbon nanotubes [133]. Electroactive substances often have good antimicrobial properties, and thus conductive gels are expected to be a good choice for addressing drug-resistant bacteria-infected wounds [134]. In addition, by capturing various signals from the human body in a non-invasive way and converting them into recognizable electrical signals for real-time monitoring, the conductive hydrogel wireless wearable sensors show broad application prospects in the field of human motion detection and medical rehabilitation. There are also some problems to overcome when using electrically responsive hydrogels to monitor wound healing in real time. For example, the sensing mechanism is relatively complex, requiring the connection of a display end and limited patient movement. Unstable circuit connections and insufficient hydrogel adhesion lead to signal interruption. The monitoring mechanism is highly sensitive, and slight touch and movement can lead to large changes in the electrical signal [135,136,137].

### 4.4. Magnetic Field Responsive Hydrogels

Magnetic fields are non-contact, easy-to-use, and easily and precisely controlled sources of stimulation. Several forms of response are programmable in magnetic hydrogel by means of an external magnetic field, such as movement, deformation, and heat production, for therapeutic purposes, without being limited by the depth of tissue penetration. Preparation methods typically involve the introduction of ferromagnetic nanoparticles (Fe_3_O_4_, CoFe_2_O_4_) or other organic ferromagnets into the three-dimensional network structure of the hydrogel by means of physical encapsulation or chemical grafting, endowing it with magnetic-responsive properties.

**Figure 7:**
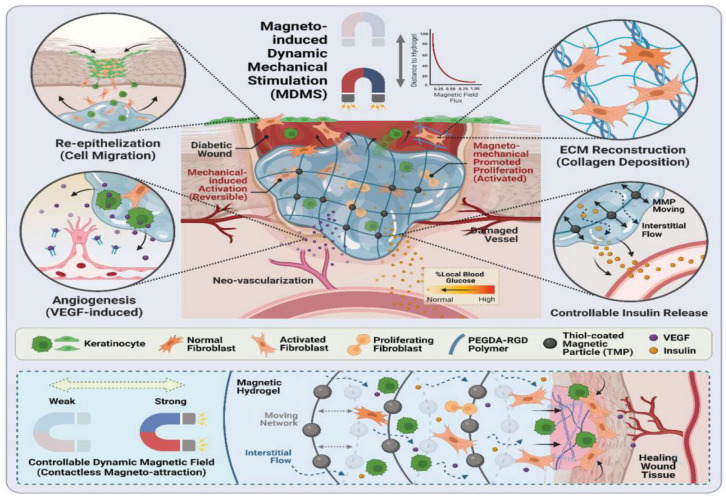
A schematic representation of magnetic hydrogel treated with magnetically induced dynamic mechanical stimulation for diabetic wound healing [108].

Magnetic nanoparticles (MNPs) can convert magnetic energy into other forms of energy under magnetic fields of different frequencies [138]. Under low-frequency magnetic fields (< 100 Hz), magnetic energy is converted into mechanical energy through dipole–dipole interactions between MNPs, and the resulting mechanical forces can deform the magnetic hydrogel and provide the necessary stimuli to cells/tissues to induce specific biological effects. It was reported after exposure to a static magnetic field that mesenchymal stem cells/stromal cells (MSCs), inoculated on magnetically responsive gelatin, have angiogenic properties that modulate the MSC secretome. Magnetic field instructions guided the alignment of MSCs and increased the expression levels of specific genes and proteins promoting angiogenesis, which were used to promote angiogenesis in tissue injuries, including bone defects, skin wounds, and cardiovascular diseases, thereby enhancing tissue regeneration. In addition, MNPs were stirred into the hydrogel network under the action of an external magnetic field, which led to the relaxation of the polymer chains, resulting in swollen and loosened polymer networks that enhanced drug diffusion [139]. On the other hand, MNPs induced mechanical vibration and the deformation of the hydrogel under the action of an external magnetic field, resulting in localized tensile and compressive stresses that stimulated drug release [140]. For example, magnetic hydrogels containing guaifenesin (GFN) have been shown to be effective for targeted drug delivery during burn wound healing [141]. Under high-frequency magnetic fields, Nair relaxation and Brownian relaxation are the heat-generating mechanisms that MNPs use to transform magnetic energy into thermal energy. Currently, hydrogels based on magnetically induced dynamic mechanical stimulation have been shown to have excellent potential for use in chronic wounds such as diabetic wounds. Reporting in the journal *Advanced Materials*, Shou et al. [108] enhanced diabetic wound healing by a factor of three through the utilization of a fully integrated, non-invasive smart bandage technology that incorporated magnetically sensitive hydrogels, cells, and wireless magnetically induced dynamic mechanical stimulation (Figure 7). They also used wound healing gel incorporating two kinds of skin cell (keratinocytes and fibroblasts) that are FDA-approved, as well as tiny magnetic particles. The mechanical stimulation of the gel, when coupled with an externally produced dynamic magnetic field, stimulates skin fibroblasts to increase their activity. According to laboratory testing, the magnetic wound healing gel promotes the production of new blood vessels, speeds up cell growth by about 2.4 times, and more than doubles collagen content. It also boosts fibroblast activity.

## 5. Multi-Responsive Hydrogels

Hydrogels that are dual-responsive or multi-responsive have garnered interest as a result of the continued investigation and development of these materials by researchers. It is possible to synergistically manage the various stages of wound healing as well as medication release by introducing two or more stimuli into a single system. This has the potential to be of utility in a variety of situations. The microenvironment of different types of skin wounds is complicated [142,143].

**Figure 8:**
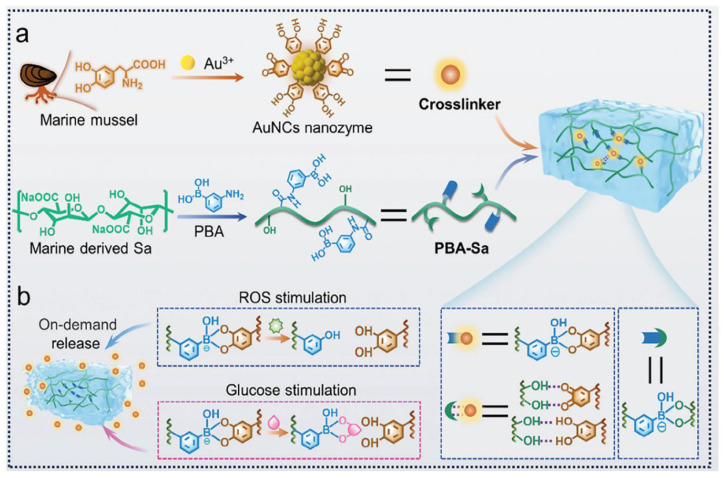
Au NCs@ PBA-Sa hydrogel preparation and dual-reaction release behavior [144].

Multi-responsive hydrogels have also been shown to have excellent potential for application in chronic wounds such as diabetic wounds. Wang et al. [144] used levodopa (L-DOPA), a bioactive small molecule derived from the marine mussel, as a ligand and reducing agent to prepare Au NCs with superoxide dismutase (SOD)-like activity (Figure 8). By forming dynamic borate bonds with phenylboronic acid-modified sodium alginate (PBA-Sa), this functional ligand not only endowed the Au NCs with significantly enhanced radical scavenging ability and excellent SOD-like enzyme activity, but also made it possible to construct Au NCs @ PBA-Sa hydrogels with significantly enhanced mechanical properties. This was accomplished by employing Au NCs. Based on the one-of-a-kind responsiveness of the borate bond to reactive oxygen species (ROS) and glucose, the hydrogel system is able to degrade and release the functional components of Au NCs in a responsive manner. This allows for the effective regulation of the harsh microenvironment of diabetic chronic wounds, which includes oxidative stress, immune dysregulation, and significant inflammation.

**Figure 9:**
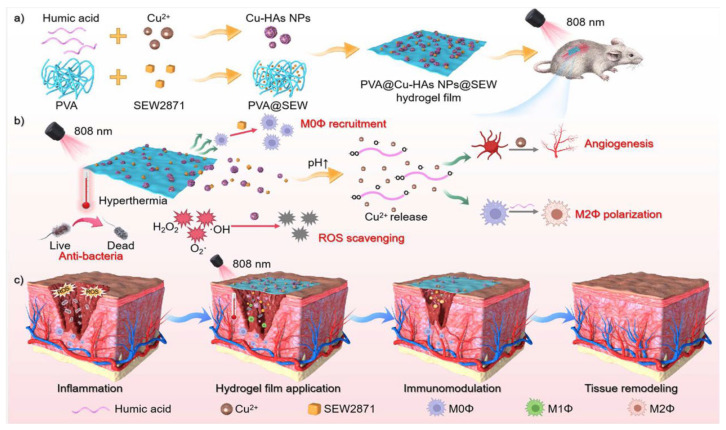
A schematic application of the PVA@ Cu-HAs NPs@ SEW hydrogel membrane to promote wound healing in bacteria-infected skin [145].

Multi-responsive hydrogels have also shown superior effects in terms of antimicrobial properties. Zha et al. [145] published an article in the journal *ACS Nano* on the preparation of near-infrared (NIR)/pH dual-responsive hydrogel thin films, with poly(vinyl alcohol) (PVA) serving as a substrate doped with humic acid (HA) and copper-ion chelated nanoparticles (Cu-HAs NPs) additionally loaded with the macrophage-recruiting agent SEW2871 (SEW), for the treatment of bacteria-infected skin wounds (Figure 9). The solubility of HAs is pH-responsive. They can form a dense structure to destroy bacteria in the early stage of the wound (acidic conditions), and then solubilize in the later stage as the pH increases to promote tissue regeneration. The Cu-HAs NPs act as a synergistic sterilizing agent that eradicates bacterial infections when irradiated with near-infrared light. The dual-responsive hydrogel film exhibits excellent antibacterial properties, with an inhibition rate of 97.06% against *Escherichia coli* (*E. coli*) and 99.42% against *Staphylococcus aureus* (*S. aureus*).

## 6. Conclusions and Outlook

Here, we take a look at how far smart-responsive hydrogels have come in helping skin wounds to heal, especially for chronic hard-to-heal wounds. Firstly, the characteristics of chronic hard-to-heal wounds, including their microenvironment, physiological changes, and therapeutic measures, are discussed in depth, with a focus on the intrinsic causes of the long healing cycle of these wounds. Subsequently, the design principles and research results of responsive hydrogels, which are capable of responding to internal stimuli (e.g., pH, blood glucose level, enzyme concentration) or external stimuli (e.g., temperature, light, electricity, and magnetic field) and achieving on-demand drug release through reversible or irreversible changes in chemical structures or physical properties, are introduced in detail. In addition, the research and development requirements, design difficulties, and outstanding achievements of multi-reaction hydrogels are also analyzed. This provides new ideas for solving the problem of chronic non-healable wounds, which are multi-stage, difficult to regulate, and highly complex.

However, this approach also has some limitations. First, chronic wounds are mostly dynamic, and it is difficult to design a smart hydrogel dressing that is multi-responsive to the entire wound healing process at the same time. Even though researchers have developed two or more responsive strategies to synergize the treatment, the interactions between the different mechanisms and the presence or absence of side effects are still issues that need to be addressed. For example, multi-reactive hydrogel systems are complex, slow to degrade, and poorly biocompatible. And it is difficult to control the release rate as a drug carrier, resulting in a slow release rate or transient release phenomenon. Second, hydrogel adhesion and peel ability need to be balanced during development, especially if the dressing needs to be changed regularly. In addition, much of the research on smart-responsive hydrogels has focused on material design, with satisfactory results in both in vitro and animal experiments, but there are many regulatory challenges for use in humans. First, the long-term safety of smart hydrogel wound dressings in the human body is a key consideration. Because wound dressings need to be in contact with the skin or wound for extended periods of time, it is important to ensure that the hydrogel material does not trigger allergic reactions, inflammatory reactions, or other adverse biological responses. This needs to be verified with long-term clinical trials and follow-up studies. Smart hydrogel wound dressings need to be biocompatible to mimic the structure and function of human soft tissue. This necessitates an in-depth study of their interaction with cells and tissues to ensure that they do not negatively affect the human body. Secondly, the effectiveness of smart hydrogel wound dressings needs to be verified through rigorous clinical trials. These trials need to prove that they have better therapeutic effects than traditional wound dressings, such as offering faster wound healing and lower infection rates. And, with the development of smart hydrogel wound dressings, it has become particularly important to develop appropriate performance standards and quality control specifications. This helps to ensure the quality and safety of products on the market and promotes the standardization of the technology. Moreover, smart hydrogel wound dressings, as medical devices, need to strictly comply with national and regional regulations related to medical devices. This includes regulatory compliance in all aspects of product design, production, registration, distribution, and use. Smart hydrogel wound dressings need to undergo a rigorous registration approval process before they are approved for human use. This includes submitting detailed registration information, undergoing evaluation by a review body, and possible on-site inspection. The complexity and time-consuming nature of the approval process may pose a challenge to the launch of the product. Therefore, the industrialization of the materials requires further clinical testing, and the impact of individual differences on therapeutic efficacy needs to be assessed.

Although there are still many challenges to overcome in optimizing the performance of hydrogels and achieving clinical translation, it is undeniable that smart-responsive hydrogels will remain a focal point in the development of wound healing dressings for skin injuries in the future. Firstly, in response to the dynamic nature of chronic wounds, intelligent hydrogel dressings should be the primary emphasis of future studies in order to develop more complex and refined multi-responsive mechanisms. This requires us to delve into the interactions of different stimulus–response mechanisms and how they synergistically promote wound healing while ensuring that there are no adverse effects. Leveraging artificial intelligence algorithms to optimize the design of response mechanisms, and utilizing machine learning models to predict environmental changes during wound healing, can guide the responsive behavior of hydrogels and achieve more personalized treatment. Secondly, there is a need to continue exploring new materials and technologies that maintain the good adhesion of hydrogels while ensuring easy removal without causing secondary damage to the wound. This may involve regulating the surface properties of hydrogels or developing novel coating technologies. Combining micro–nano bionic technology to design hydrogels with micro structured surfaces can enhance their adhesion to wounds while facilitating easy removal during dressing changes. The clinical trial process for smart-responsive hydrogels, particularly regarding drug release efficacy and mechanism responses in humans, should be accelerated to obtain more reliable data, thus supporting their safety and effectiveness. For example, the preparation conditions of smart hydrogels are more demanding, and the synthesis is more complex, involving the control of temperature, the control of time, and many other fine steps, which will increase the cost of large-scale production. It is difficult to control the consistency and stability of the performance, including biocompatibility and degradability, and the yield of each batch, which requires a strict quality monitoring system. In addition, a larger scale of production requires a larger range of biological toxicity evaluation to ensure safety and efficacy. Furthermore, utilizing big data analytics and artificial intelligence to assess the impact of individual differences (such as age, gender, genetic background, etc.) on the therapeutic effect of smart hydrogels can provide a basis for personalized medicine.

When it comes to treating chronic, hard-to-heal wounds, smart-responsive hydrogels show great promise, but breakthroughs in multiple response mechanisms, adhesion and peelability, clinical testing, and individual difference assessment are needed to fully realize their potential. Through interdisciplinary collaboration and technological innovation, it is expected that smart-responsive hydrogels will be developed into advanced medical materials for integrated diagnosis and treatment, providing more efficient and personalized treatment solutions for chronic wound patients.

## Figures and Tables

**Table 1:** Summary of internal stimulus-responsive hydrogels applied to skin wound healing.

Stimulation	Hydrogel	Crosslink	Animal Model	Ref.
pH	CMCS/PVP/TA multifunctional hydrogel	Hydroge-bonding, electrostatic interactions	Mouse whole-skin defect model	[58]
	PVA–borax/resveratrol grafted cellulose nanofiber hydrogel	Dynamic borate bonding, hydrogen bonding	Aureus-infected female KM mouse model	[59]
	in situ Ag NP–silk microfiber–hydrazide hyaluronic acid hydrogel	Dynamic acyl hydrazone bonds, metal chelation	Mouse back burn model	[60]
	Pluronic F127/carbon nanotubes/N-carboxyethyl chitosan hydrogel	Dynamic Schiff base bonds, Π–Π covalency, hydrophobic interactions	Mouse whole-skin wound infection model	[61]
ROS	SPBA/L-Arg/PVA hydrogel	Hydrogen bonding	Mouse whole-layer skin defect model	[62]
	NHS-TK-NH (ROS cleavable joint)/RSG-CCL22 hydrogel	Amide bond, carbon-carbon covalent	Mouse whole-layer diabetic wound model	[63]
	Polyvinyl alcohol–TPA (ROS response connector) hydrogel	Phenylboronic acid ester bond	Bacteria-infected diabetic mouse wound model	[64]
	Copper-tannic acid (CuTA) nanosheet–GelMA hydrogel	Free radical polymerization, hydrogen bonding, borate bonding	Rat diabetic wound model	[65]
Enzyme	Pluronic F-127–methylcellulose hydrogels loaded with GT/siMMP9	Hydrogen bonding	Rat diabetic wound model	[66]
	supramolecular peptide hydrogels doped with nanoparticles	Self-assembly, electrostatic interactions	Rat diabetic wound model	[67]
	Injectable and microporous fibrous gel particles containing bioglass and siRNAs	Photocrosslinking	Rat diabetic wound model	[68]
	Phenylboronic acid-grafted quaternized chitosan/polyvinyl alcohol hydrogels	Boronate bonds	Rat diabetic wound model	[69]
Glucose	Lipoic acid-modified chitosan injectable hydrogel	Photocrosslinking, amide reaction	Diabetic wound model in mice	[70]
	Ag NPs/phenylboronic acid grafted onto hyaluronic acid–glycol chitosan hydrogels	Dynamic borate bonding	Bacterially infected diabetic rat wound model	[71]
	Phenylboronic acid-modified linseed gum–polyvinyl alcohol hydrogels loaded with PMX, L-ascorbic acid, CA	Dynamic borate bonds, hydrogen bonds	Whole-layer wound healing model of dorsal skin defects in type I diabetic male Kunming mice	[72]
	Polyethylene glycol copolymerized (glycerol sebacate)/dihydrocaffeic acid and l-arginine-copolymerized chitosan hydrogel	Schiff base bonds, phenyl borate bonds	Whole-layer model of foot skin defect wound healing in type II diabetic SD rats	[73]

**Table 2:** Summary of external stimulus-responsive hydrogels applied to skin wound healing.

Stimulation	Hydrogel	Crosslink	Animal Model	Ref.
Temperature	Amphiphilic ion-based carboxymethyl cellulose hydrogels	Hydrogen bonding	*Staphylococcus aureus*-infected mouse wound model	[97]
	VEGF-loaded N-acryloylglycinamide/1-vinyl-1,2,4-triazole/ PNIPAM polymer hydrogels	Hydrogen-bonding, non-covalent interactions	Rat diabetic wound model	[98]
	Poly(citrate-glycol-siloxane)-based molecular hybridized hydrogels	Hydrogen bonding	MRSA-infected mouse wound model	[99]
	Multilayered micellar polyarc complex hydrogels loaded with Ag NPs	Ionic interactions, Steglich esterification reactions	Rat total skin defect model	[100]
Photo	DA-grafted hyaluronic acid–PDA-coated Ti_3_C_2_/MXene nanosheets for injectable hydrogels	Oxidative coupling, amide bonds	Diabetic mouse whole-layer skin defect model	[101]
	Polydopamine-coated cellulose nanocrystals/phenylazo-capped Pluronic F127-quaternized chitosan-grafted cyclodextrin hydrogels	Schiff base bonding, hydrogen bonding	Rat total skin defect model	[102]
	Bimetallic CuCo-doped nitrogen–carbon nano-enzyme functionalized hydrogels	Fenton reaction, oxidative coupling	MRSA mouse wound infection model	[103]
	Gelatin-based smart three-dimensional hydrogels	Amide bonds, hydrogen bonds, electrostatic interactions and aromatic π–π interactions	Rat total skin defect model	[104]
Electric	Odium alginate oxide-carboxymethyl chitosan injectable hydrogel	Dynamic imine bonding, in situ photoreduction	New Zealand white rabbit whole-skin defect model	[105]
	Polypyrrole-F127 composite hydrogel	Electrochemical crosslinking	Mouse whole-layer skin defect model	[106]
	Sensor hydrogel based on amphiphilic ionic thermal glucose-sensitized skin-like sensitization	π–π interactions, hydrogen bonding	Diabetic mouse whole-skin defect model	[107]
Magnetic	Poly(ethylene glycol) diacrylate-based hydrogels loaded with RGD and TMP	Alkene reaction	Diabetic mouse whole-layer skin defect model	[108]
	MXene-encapsulated MNP-based dual-network hydrogels	Covalent crosslinking, ionic crosslinking	Infected diabetic rat whole-layer skin defect model	[109]
